# Overview of the Structural, Electronic and Optical Properties of the Cubic and Tetragonal Phases of PbTiO_3_ by Applying Hubbard Potential Correction

**DOI:** 10.3390/ma16124302

**Published:** 2023-06-10

**Authors:** Issam Derkaoui, Mohamed Achehboune, Roberts I. Eglitis, Anatoli I. Popov, Abdellah Rezzouk

**Affiliations:** 1 Laboratory of Solid State Physics, Faculty of Sciences Dhar El Mahraz, University Sidi Mohammed Ben Abdellah, P.O. Box 1796, Fez 30000, Morocco; derkaouiissam@gmail.com (I.D.); rezzouk@yahoo.fr (A.R.); 2 Laboratoire de Physique du Solide, Namur Institute of Structured Matter, University of Namur, Rue de Bruxelles 61, 5000 Namur, Belgium; achehboune.mohamed01@gmail.com; 3 Institute of Solid State Physics, University of Latvia, 8 Kengaraga Str., LV1063 Riga, Latvia; rieglitis@gmail.com

**Keywords:** PbTiO_3_, GGA+U, Hubbard correction, electronic properties, chemical bonds, optical properties

## Abstract

We have performed a systematic study resulting in detailed information on the structural, electronic and optical properties of the cubic (*P*m$\bar{3}$m) and tetragonal (*P*4mm) phases of PbTiO_3_ applying the GGA/PBE approximation with and without the Hubbard U potential correction. Through the variation in Hubbard potential values, we establish band gap predictions for the tetragonal phase of PbTiO_3_ that are in rather good agreement with experimental data. Furthermore, the bond lengths for both phases of PbTiO_3_ were assessed with experimental measurements, confirming the validity of our model, while chemical bond analysis highlights the covalent nature of the Ti–O and Pb–O bonds. In addition, the study of the optical properties of the two phases of PbTiO_3_, by applying Hubbard’ U potential, corrects the systematic inaccuracy of the GGA approximation, as well as validating the electronic analysis and offering excellent concordance with the experimental results. Therefore, our results underline that the GGA/PBE approximation with the Hubbard U potential correction could be an effective method for obtaining reliable band gap predictions with moderate computational cost. Therefore, these findings will enable theorists to make use of the precise values of these two phases’ gap energies to enhance PbTiO_3_’s performance for new applications.

## 1. Introduction

Perovskite-type ABO_3_ (A = Ba, Sr, Pb) oxides’ crystal structure as well as their physical and optical characteristics have been thoroughly explored recently, owing to their widespread development and their functional properties suitable for many technological applications [1,2,3]. In particular, PbTiO_3_ itself has been frequently and intensively studied due to its excellent physical properties, which makes it a promising material for a variety of applications, including ferroelectric memory cells [4,5], optoelectronic devices [6,7], dielectric capacitors [8], electromechanical devices [9,10] and pyroelectric applications [11].

Up to now, PbTiO_3_’s cubic (*P*m$\bar{3}$m) and tetragonal (*P*4mm) phases have been among the most experimentally investigated structures [12,13,14,15]. Indeed, perovskite PbTiO_3_ exhibits a ferroelectric–paraelectric phase transition at 763 K. For this reason, a study of the temperature-dependent crystal structure is needed to understand the precise mechanism of ferroelectricity in this material [16,17]. Ferroelectricity is created when cations and anions move away from their equilibrium locations, creating spontaneous dipole moments. The tetragonal phase possesses a non-centrosymmetric space group *P*4mm and is polar and ferroelectric at low temperatures [18,19]. The spontaneous polarization diminishes as the temperature rises, which causes the crystal structure to transition into a paraelectric cubic phase [6,19].

Density functional theory (DFT) calculations furnish a useful tool for calculating the crystal structure, electronic and optical characteristics of materials [20,21,22,23,24]. In the DFT calculations used for the band gap computations of PbTiO_3_, both the local density approximation (LDA) and the generalized gradient approximation (GGA) are the most widely used exchange correlation energy functionals [25,26]. So far, the band gap of the cubic phase of PbTiO_3_ is unknown experimentally, while the DFT calculations of the band gap of the tetragonal phase of PbTiO_3_ do not show the same results as the experimental investigations, which still need to be improved. In accordance with some previous experimental studies, the Γ–Γ band gap of the tetragonal phase (*P*4mm) of PbTiO_3_ is equal to 3.40 eV [14,15]. By contrast, the calculated band gap values obtained from conventional DFT computations are 1.723 eV (LDA) [25] and 1.68 eV (GGA/PBE) [26] for the cubic phase and 1.56 eV (LDA) [27] and 1.47 eV (LDA) [28] for the tetragonal phase. They considerably underestimate the experimental band gap value, and therefore these approaches are insufficient to accurately describe the electronic structure of PbTiO_3_, which significantly influences its optical characteristics.

To enhance the accuracy of the calculations of the electronic structure and the optical properties, as well as to obtain better results in accordance with the actual data, we have employed the modified Hubbard U potential for the Pb–5d, Ti–3d and O–2p orbitals. Actually, the Hubbard U potential is widely used as a fitting parameter, determined in a semi-empirical way by looking for values that reproduce the experimental results, such as the band gap of a given material. Particularly strongly correlated electronic states may be described using Hubbard’s U addition to the usual GGA technique, and this is done by isolating a few degrees of freedom that are pertinent to the correlation [29,30]. Additionally, several studies have shown that altering the Hubbard U parameters may enhance the band gap of the majority of materials [21,22,23,31].

One of the primary goals of this study is to give a good prediction for both the electronic structure and the optical properties of PbTiO_3_. We will first investigate the GGA approach with and without the Hubbard U potential correction, for a comparative study of the two approximations. Second, to overcome the limitations of the GGA approximation so as to achieve good concordance with the experimental results, we will implement the Hubbard U correction in our simulations. Hence, our calculations offer a good reference in terms of electronic and optical properties of both the cubic and tetragonal phases of PbTiO_3_, which will allow scientists to apply different external parameters (e.g., doping) to this material to study its properties, employing a gap energy that is exactly in line with the one obtained through experiment.

This original work first presents a detailed overview of the geometry optimization to identify the most appropriate plane wave pseudopotential methods, k-points and cut-off energies for both the cubic and tetragonal phases of PbTiO_3_ using the GGA approximation, followed by a thorough examination of the Hubbard correction. In addition, the chemical bonds of PbTiO_3_ were studied with the adopted Hubbard potential parameters (O–2p, Ti–3d and Pb–5d). Subsequently, a comparative study with and without Hubbard U correction to analyze the electronic and optical properties of PbTiO_3_ is also presented, followed by the conclusions of this work.

## 2. Computational Model

In the present calculations, we employed the Cambridge Serial Total Energy Package module (CASTEP) [32], which is based on density functional theory (DFT) with the generalized gradient approximation of Perdew–Burke–Ernzerhof [33,34] (GGA-PBE) for exchange-correlation potential. To analyze the interactions involving the valence electrons and ionic core, the valence electron configurations of [Xe] 6s^2^4f^14^5d^10^6p^2^ for Pb, [Ar] 3d^2^4s^2^ for Ti and [He] 2s^2^2p^4^ for O were used. Additionally, self-consistent energy convergence thresholds of 10^−5^ eV/atom for total energy, 0.03 eV/ for maximum force, 0.05 GPa for maximum stress and 0.001 for maximum displacement were used to optimize the geometry.

Furthermore, we have systematically examined the dielectric function of PbTiO_3_, which is defined as follows:

(1) $$\varepsilon (\omega )=\varepsilon_{1}(\omega )+i\varepsilon_{2}(\omega )$$

where ω is the incident photon frequency, and $\varepsilon_{1}(\omega )$ and $\varepsilon_{2}(\omega )$ denote the real and imaginary parts of the dielectric function, respectively. To calculate $\varepsilon_{2}(\omega )$, all transitions from occupied to unoccupied states are summed by the following relation [35]:

(2) $$\varepsilon_{2}(\omega )=\frac{2\pi e^{2}}{\Omega \varepsilon_{0}}{\sum_{k,v,c}<\Psi_{k}^{c}|\hat{u}.r|\Psi_{k}^{v}>}^{2}\delta (E_{k}^{c}-E_{k}^{v}-\hbar \omega )$$

In which *e*, $\varepsilon_{0}$, Ω and ℏω designate the electronic charge, the vacuum dielectric constant, the volume and the energy of the incident phonon, respectively. $\Psi_{k}^{c}$ and $\Psi_{k}^{v}$ are the wave functions of the conduction band (CB) and the valence band (VB) at the k-point, respectively. The vector that indicates how the incident electric field is polarized is called $\hat{u}$. The momentum operator is $\hat{u}.r$ and the subscripts *c* and *v* denote the CB and VB, respectively [35]. The Kramers–Kronig relation [35] could be used to determine the values of $\varepsilon_{1}(\omega )$:

(3) $$\varepsilon_{1}(\omega )=1+\frac{2}{\pi }P\int_{0}^{\infty }\frac{\varepsilon_{2}(\omega^{\prime })\omega^{\prime }}{\omega^{\prime }{}^{2}-\omega^{2}}d\omega$$

where *P* is the integral’s primary value.

Hence, the optical properties using the GGA/PBE + U approximation without the scissor correction, including the optical absorption coefficient (*α*(*ω*)), reflectivity (*R*(*ω*)), refractive index (*n*(*ω*)), extinction coefficient (*k*(*ω*)), as well as the energy loss function (*L*(*ω*)) were calculated through the complex dielectric function (*ε*(*ω*)) and are formulated as follows [35]:

(4) n(ω)=12[ε1(ω)2+ε2(ω)2+ε1(ω)]12

(5) k(ω)=12[ε1(ω)2+ε2(ω)2−ε1(ω)]12

(6) L(ω)=ε2(ω)[ε1(ω)2+ε2(ω)2]

(7) $$R(\omega )={\left|\frac{\sqrt{\varepsilon_{1}+i\varepsilon_{2}}-1}{\sqrt{\varepsilon_{1}+i\varepsilon_{2}}+1}\right|}^{2}$$

(8) α(ω)=2ωC0[ε12(ω)+ε22(ω)2−ε1(ω)]12

where *C*_0_ is the vacuum-state speed of light.

In the geometry optimization section, we will first identify the most appropriate plane wave pseudopotential methods, k-points and cut-off energies to obtain the equilibrium lattice parameters for both the cubic and tetragonal phases of PbTiO_3_ with minimal deviations from the experiment via the GGA/PBE approximation. Then, we will search through the Hubbard U potential for the parameters that reproduce the gap energies in perfect agreement with the experimental results.

## 3. Results and Discussion

### 3.1. Geometry Optimization

#### 3.1.1. Appropriate Pseudopotential Methods

**Figure 1:**
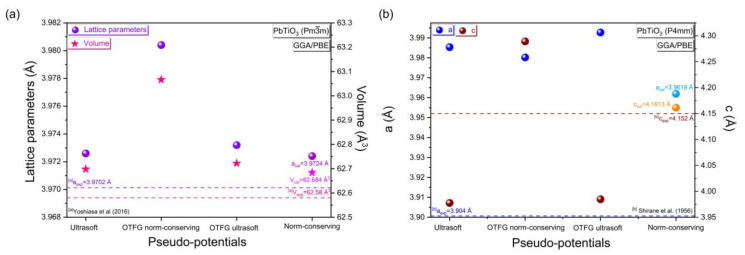
Calculation and comparison with the experimental results as a function of the variation in the pseudopotential methods of (**a**) the lattice parameters and volume of the cubic phase of PbTiO_3_, (**b**) the lattice parameters (a, c) of the tetragonal phase of PbTiO_3_; the k-point and cut-off energy values were fixed at 2 × 2 × 2 and 500 eV, respectively, using the GGA/PBE approximation. For both the cubic and tetragonal phases of PbTiO_3_, the pale colors of the lattice parameters and volume indicate that the norm-preserving pseudopotential method has the closest relative deviation to the experimental value. ^[a]^ Experimental data from Yoshiasa et al [12]; ^[b]^ Experimental data from Shirane et al. [13].

Figure 1 shows the lattice parameters and volume calculated in comparison with the experimental results using the GGA/PBE approximation with different pseudopotential methods for the cubic (*P*m$\bar{3}$m) and tetragonal (*P*4mm) phases of PbTiO_3_. While the detailed calculation of the lattice parameters, c/a tetragonality and volume deviations are presented in the Supplementary Materials Section (Table S1).

To begin with, we will investigate the appropriate plane wave pseudopotential methods (the ultrasoft, the OTGF ultrasoft, the OTGF norm-conserving and the norm-conserving) for the cubic (*P*m$\bar{3}$m) and tetragonal (*P*4mm) phases of PbTiO_3_. For the cubic phase, when we compare with the experimental data [12], the relative deviations of lattice parameters between the calculated structure parameters and the experimental values are about 0.060% for the ultrasoft, 0.075% for the OTGF ultrasoft, 0.256% for the OTGF norm-conserving and 0.055% for the norm-conserving pseudopotential methods. In the case of the tetragonal phase (*P*4mm) of PbTiO_3_, the results presented in Figure 1b show that the optimized lattice parameters (a, c) provide minimal deviation from the experiment [13] and this is achieved by the norm-conserving pseudopotential method. Hence, the lattice parameters for both the cubic and tetragonal phases of PbTiO_3_ calculated by the norm-conserving pseudopotential method display the closest relative deviation to the experimental value.

#### 3.1.2. Appropriate k-Points and Cut-Off Energy

**Figure 2:**
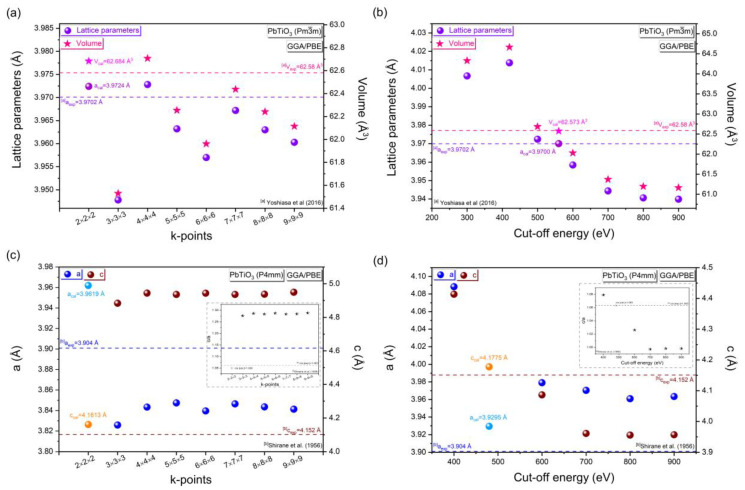
Calculation and comparison with the experimental results as a function of the k-point and cut-off energy value variation in (**a**,**b**) the lattice parameters and volume of the cubic phase of PbTiO_3_, (**c**,**d**) the lattice parameters (**a**,**c**) with c/a tetragonality of the PbTiO_3_ tetragonal phase. For the cubic phase, the distinct colors of the lattice parameters and volume indicate that the k-point and cut-off energy values with the closest relative deviation from the experimental data are 2 × 2 × 2 and 560 eV, respectively. In the case of the tetragonal phase, the pale colors of the lattice parameters (**a**,**c**) indicate that the values of the k-points and the cut-off energy that have the closest relative deviation from the experimental results are 2 × 2 × 2 and 480 eV, respectively. ^[a]^ Experimental data from Yoshiasa et al [12]; ^[b]^ Experimental data from Shirane et al. [13].

In an attempt to obtain the best minimum deviation from the experimental and optimized lattice parameters, c/a tetragonality, and volume of the cubic (*P*m$\bar{3}$m) and tetragonal (*P*4mm) phases of PbTiO_3_, we will investigate the most convenient k-point and cut-off energy values. The pristine cell parameters a = b = c = 3.9702 Å (α = 90°; volume = 62.58 Å) [12] and a = b = 3.904 Å, c = 4.152 Å (α = 90°; volume = 63.28 Å; tetragonality c/a = 1.063) [13] were originally used for constructing the cubic (*P*m$\bar{3}$m) and tetragonal (*P*4mm) phases of PbTiO_3_, respectively. Initially, by keeping the energy cut-off value constant (e.g., 500 eV), we will change the grid values (k-points). Then, after getting an appropriate value of k-points, we will maintain this value constant and vary the cut-off energy to get the most appropriate cut-off energy value. Additionally, further calculation of lattice parameters and volume deviations as a function of varying k-point and cut-off energy values is presented in Tables S2–S5 in the Supplementary Materials Section. In general, the k-points in electronic structure theory are sampling points in the first Brillouin zone of the material, and this set of discrete points in the unit cell allows defining energy values such as energy bands. Setting the k points to 2 × 2 × 2 results in the smallest difference between the experimental and optimized volume and lattice parameters for the cubic phase (Figure 2a coupled with Table S2). Furthermore, following the use of several cut-off energies, we have found that the minimum deviation of the lattice parameters and volume is −0.005% and −0.011%, respectively, occurring for the cut-off energy of 560 eV (Figure 2b coupled with Table S4). On the other hand, for the tetragonal phase, the smallest divergence between the experimental and optimized lattice parameters a and c, as well as c/a tetragonality, occurs by setting the k-points to 2 × 2 × 2 (Figure 2c coupled with Table S3). Furthermore, after using several cut-off energies, we found that the minimum deviation of a and c for the cut-off energy of 480 eV (Figure 2d coupled with Table S5) is less than 0.65%. Thus, there is good agreement between the optimized tetragonality c/a and the experimental results.

Table 1 summarizes the most suitable k-point and cut-off energy values of the cubic (*P*m$\bar{3}$m) and tetragonal (*P*4mm) phases of PbTiO_3_ using the GGA/PBE approximation. These outcomes highlighted that the difference in lattice parameters between computed and conventional data [12,13] was less than 0.0051% and 0.65% for the cubic (*P*m$\bar{3}$m) and tetragonal (*P*4mm) phases, respectively, which allows us to investigate the performance and diversity of structural parameters of PbTiO_3_. Hence, the cut-off and k-point values selected after the optimization are in good agreement and demonstrate the validity of our model, together with the smallest relative deviation from the experimental data. Furthermore, in Table 2, a more extensive comparison between our optimized lattice parameters (a, c and c/a tetragonality) and those obtained experimentally [12,13,36,37] and theoretically [25,26,28,38,39,40], for both the cubic and tetragonal phases of PbTiO_3_ is presented. After the geometric optimization of the cubic (tetragonal) phase of PbTiO_3_, we obtained a minimal deviation between the optimized and experimental lattice parameters, when using the norm-conserving pseudopotential method with a 2 × 2 × 2 (2 × 2 × 2) k-point value and a cut-off energy of 560 eV (480 eV). The next step is to perform a good prediction of the electronic structure of the two phases of PbTiO_3_ using the Hubbard U correction.

#### 3.1.3. Hubbard Potential Correction

For both the cubic and tetragonal phases of PbTiO_3_, it should be noted that the Hubbard U potential of the Pb–5d orbital does not affect the variation in gap energy, owing to its position in the internal electronic structure close to the VB. Furthermore, we demonstrate in the Supplementary Materials Section (Tables S6 and S7) that the gap energy is not influenced even when varying the Hubbard potential values for Pb–5d electrons. To precisely characterize the electronic structures, we used the modified Hubbard U-potential to the Ti–3d and O–2p electrons. The band gaps for the cubic and tetragonal phases of PbTiO_3_ using simply the GGA/PBE approximation in the spin polarization domain and without the Hubbard correction are 2.207 eV and 2.213 eV, respectively (Table 3). This difference in band gap shows that the ions (Ti and O) under consideration have strongly correlated electrons, which in turn necessitates that we investigate the Hubbard U correction in our calculations.

**Figure 3:**
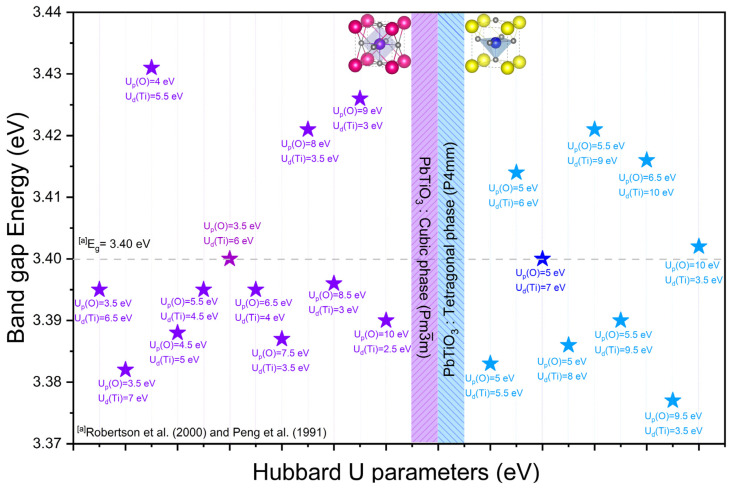
Comparison between the calculated band gaps of the cubic and tetragonal phases of PbTiO_3_ with experimental data highlighting the Hubbard U parameters chosen for the O–2p and Ti–3d orbitals; the value of U_d_(Pb) has been fixed at 0 eV. For the cubic and tetragonal phases of PbTiO_3_, the distinct colors of the Hubbard U parameters (U_p_(O) = 3.5 eV and U_d_(Ti) = 6 eV for the cubic phase, and U_p_(O) = 5 eV and U_d_(Ti) = 7 eV for the tetragonal phase) result in a gap energy in perfect agreement with the value obtained experimentally. The star symbols symbolize the Hubbard U parameters used for U_p_(O) and U_d_(Ti), with U_d_(Pb) kept at 0 eV. ^[a]^ Experimental data from Robertson et al. [14] and Peng et al. [15].

Following the probability series related to the choice of the Hubbard U potential for the Ti–3d and O–2p electrons (see Tables S6 and S7), we have presented in Table 3 and Figure 3 the influence of the chosen Hubbard U parameter (i.e., with minimal deviations) on the band gaps obtained from the tetragonal and cubic phases of PbTiO_3_. Experimentally, the tetragonal phase of PbTiO_3_ displays a band gap of 3.40 eV at the Γ–point [14,15], while the band gap of the cubic phase of PbTiO_3_ is unknown experimentally. As we can see from Table 3, we fixed the Hubbard U parameters for the O–2p and Ti–3d electrons at 5 eV and 7 eV, and 3.5 eV and 6 eV, for the tetragonal and cubic phases, respectively. This resulted in a gap energy that is exactly 3.400 eV, in conformity with the experimental findings. Moreover, our results also allow us to estimate most of the calculated gap energies which are close to the energies obtained experimentally for the tetragonal phase of PbTiO_3_. In what follows, we will use the Hubbard parameters U_p_(O) = 5 eV, U_d_(Ti) = 7 eV and U_d_(Pb) = 0 eV for the tetragonal phase and U_p_(O) = 3.5 eV, U_d_(Ti) = 6 eV and U_d_(Pb) = 0 eV for the cubic phase to analyze the chemical bonds of PbTiO_3_, including bond lengths, Mulliken charges, effective valence charges and electron charge density. Then, we will also perform a comparative study with and without the Hubbard U correction to investigate the electronic and optical properties of the two phases of PbTiO_3_.

### 3.2. Chemical Bonds

#### 3.2.1. Bond Lengths, Mulliken Charges and Effective Valence Charges

**Figure 4:**
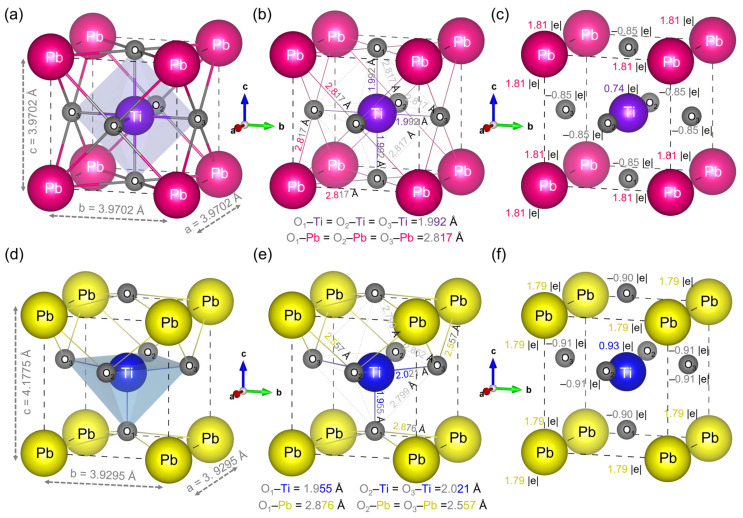
(**a**,**d**) Schematic of the crystalline structure of the primitive cell of the cubic and tetragonal phases, respectively, (**b**,**e**) bond lengths and (**c**,**f**) Mulliken charges (where “e” represents the elementary charge) of the cubic and tetragonal phases of PbTiO_3_, respectively, using the GGA/PBE + U approximation.

The cubic and tetragonal phases of the perovskite-like substance PbTiO_3_ (with five atoms in the primitive cell) were the focus of this study. In the cubic (*P*m$\bar{3}$m) phase with local symmetry $O_{h}^{1}$, the titanium is positioned at the origin of a cubic unit cell, the lead is positioned at the corners of a cube outside the oxygen octahedron, and the oxygen is centered on the face of a cubic unit cell (Figure 4a). Meanwhile, in the tetragonal phase (*P*4mm) local symmetry $C_{4v}^{1}$, the titanium atom is located in the center of the unit cell, surrounded by six oxygen atoms, and the lead atoms share each of the corners (Figure 4d).

To determine the bonding nature of Pb–O and Ti–O and to validate our theoretical results based on experimental data, the calculated bond lengths, Mulliken charges and effective valence charges of the cubic and tetragonal phases of PbTiO_3_ with GGA/PBE + U approximation are presented in Figure 4b,c,e,f coupled with Table 4. In the case of the cubic phase, the bond lengths of Pb–O, Ti–O and O–O are 2.817 Å, 1.992 Å and 2.817 Å, respectively. Comparing with the experimental values of the cubic phase of PbTiO_3_ obtained by Shirane et al., which are 2.80 Å and 1.98 Å for the Pb–O and Ti–O bond distances, respectively [13], as well as with the experimental data obtained by Grazer et al., which are 2.81 Å and 1.99 Å for the Pb–O and Ti–O bond distances, respectively [36], our results are in good agreement with these experimental results as well as with the theoretical calculation (e.g., Ti–O = 1.990 Å) [26]. Furthermore, for the tetragonal phase, the bond lengths of Pb–O_1_, Pb–O_2_, Ti–O_1_ and Ti–O_2_, are 2.876 Å, 2.557 Å, 1.955 Å and 2.021 Å, respectively (Figure 4e), which agrees well with the experimental data [13].

The Mulliken charge in such a crystal lattice describes the amount of sheared electron density of an atom, with greater positive values indicating that the atom in question contributes greater numbers of electrons; the effective valence represents the difference with the formal ionic charge. The growing covalency shows that the values remain away from zero and perfectly ionic bonding indicates that the effective valence value equals 0. As we can see from Table 4, for the cubic (tetragonal) phase, the effective valence charges calculated are 1.81 e (1.57 e) and 10.0 e (10.0 e) for Ti and Pb cations, respectively, suggesting that the Ti–O and Pb–O bonds are covalent in nature, where, Ti–O and Pb–O have partial and strong covalent bonding, respectively.

#### 3.2.2. Electron Charge Density

**Figure 5:**
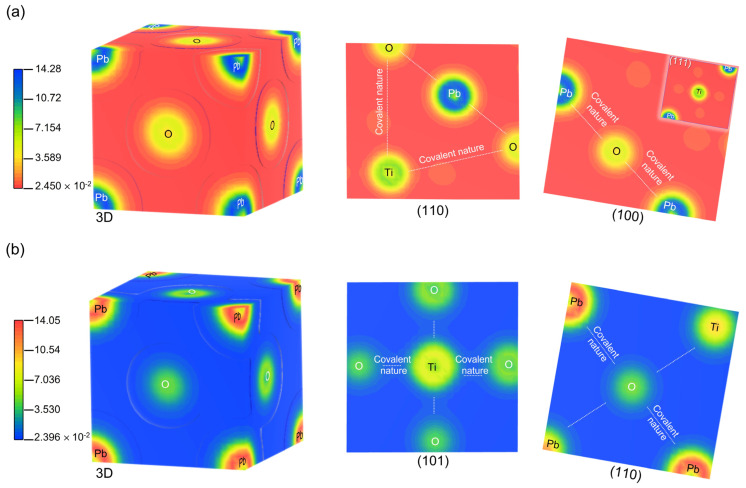
Electron density distribution maps in (3D) and (2D) in the most significant crystallographic planes of PbTiO_3_ for (**a**) the cubic phase and (**b**) the tetragonal phase, respectively. For the cubic phase, the blue and green regions surrounding the Pb or Ti atom indicate electron enrichment, while the yellow and orange regions indicate electron loss. In the case of the tetragonal phase, this is reversed.

Difference in electron density mappings, calculated with the GGA/PBE + U approximation against the superposition density for Pb^2+^, Ti^4+^ and O^2−^ ions, were graphed in the most important crystallographic planes and shown in Figure 5. The scales on the left side of the figure represent the amount of the electronic density between Pb, Ti and O ions, indicating the corresponding high electron density at high values, while with decreasing values the electron distribution progressively vanishes. In the most significant crystallographic planes (110) and (100) for the cubic phase, and (101) and (110) for the tetragonal phase of PbTiO_3_ we can see electron density distributions between Ti and O that show the electrons partially overlapping each other, partially supporting the covalent character between the oxygen and titanium atoms. In addition, we can discriminate a strong significant electron overlap between Pb and O, indicating the strong covalent nature between lead and oxygen. These findings support the covalent bonding action between Ti–O and Pb–O outlined above and are compatible with the literature currently in use [25,39].

### 3.3. Electronic Properties

#### Band Structure and Density of States

**Figure 6:**
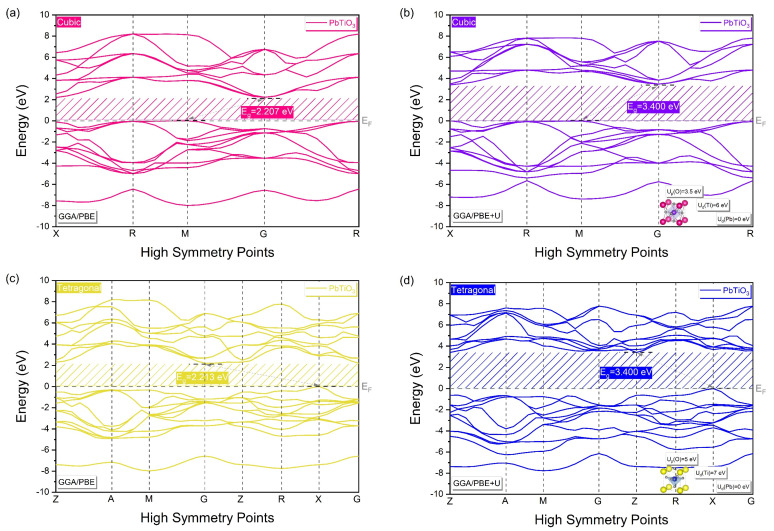
Band structure of PbTiO_3_ without and with Hubbard potential correction, (**a**,**b**) for the cubic phase, (**c**,**d**) for the tetragonal phase. Each band energy is shifted with reference to the Fermi level, being set to zero.

Figure 6 illustrates the computed band structures in high-symmetry directions in the Brillouin Zone (BZ) for the cubic and tetragonal phases of PbTiO_3_ using the GGA/PBE approximation with and without Hubbard U correction.

By applying the GGA/PBE approximation only (Figure 6a,c), an indirect band gap of 2.207 eV and 2.213 eV at the M–G and X–G points is shown as characteristic of the band structure of the cubic and tetragonal phases of PbTiO_3_, respectively. However, this obtained gap energy value is still much lower than the experimentally obtained one (3.40 eV). Thus, for overcoming the limitations of the GGA, Hubbard U correction was added to the GGA/PBE approximation, as discussed before, resulting in a band gap of 3.400 eV (Figure 6b,d), which is in quite good agreement with the experimental results [14,15]. Because of this, the accuracy of our calculations following optimization and the selection of Hubbard parameters for the cubic and tetragonal phases supports the applicability of the used model.

**Figure 7:**
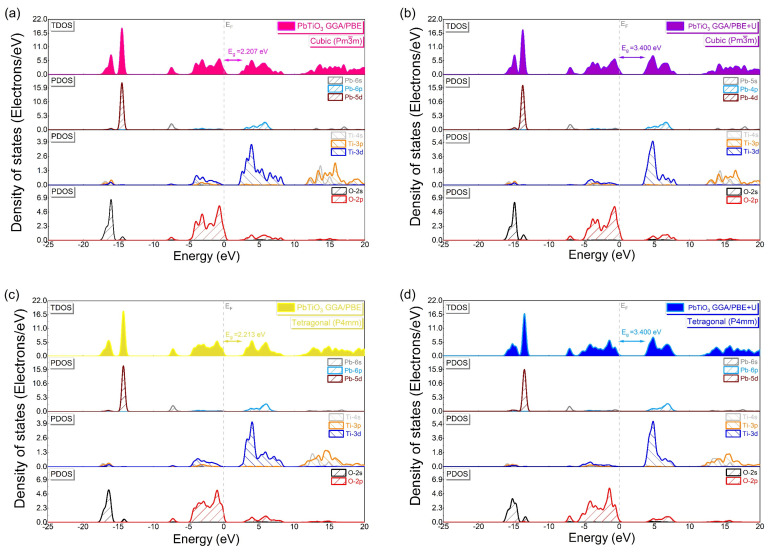
Total and partial density of states of PbTiO_3_ without and with Hubbard potential correction (**a**,**b**) for the cubic phase and (**c**,**d**) for the tetragonal phase.

**Figure 8:**
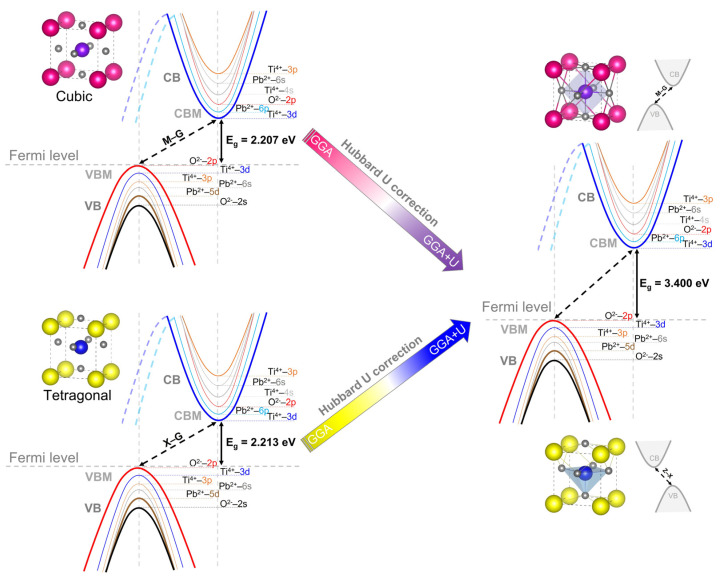
Schematic of the electronic orbital distribution of the cubic and tetragonal phases of PbTiO_3_, without and with Hubbard potential correction. The colors used for illustrating the distribution of electronic orbitals in the cubic and tetragonal phases of PbTiO_3_ are similar to those used in the PDOS study.

We computed the total (TDOS) and partial (PDOS) densities of states along with a diagram of the electronic orbital distributions of the cubic and tetragonal phases of PbTiO_3_ in order to understand the electronic properties, and in particular the atomic orbital contributions in the development of each energy band (Figure 7). With and without the Hubbard correction, the PDOS analysis shows the same electronic contributions (Figure 7a–d) where the VB consists predominantly of contributions from the 2p and 2s orbitals of oxygen and the 5d orbitals of lead, with minor contributions from the 3d and 6s orbitals of titanium and lead, respectively. The 3d titanium and 6p lead orbitals make up the majority of the CB, whereas the 2p oxygen orbitals make up a small portion of it. In addition, the 3p and 4s titanium orbitals contribute to the conduction bands at higher energies. Figure 8 illustrates this graphically, showing an indirect band gap at the M–G and X–Z points for the cubic and tetragonal phases, respectively, in which the top of the VBs is created by the O 2p orbitals as the bottom of the CBs is formed by the Ti 3d orbitals. From Figure 7, it is evident that the application of the Hubbard U potential correction leads to an increase in the intensity of the 3d orbital of titanium, accompanied by a displacement of 1.187 eV of the CB toward higher energies, which allows restoring the experimental band gap of the tetragonal phase (3.40 eV) [14,15] and this compensates for the systematic error of the GGA approximation.

### 3.4. Optical Properties

**Figure 9:**
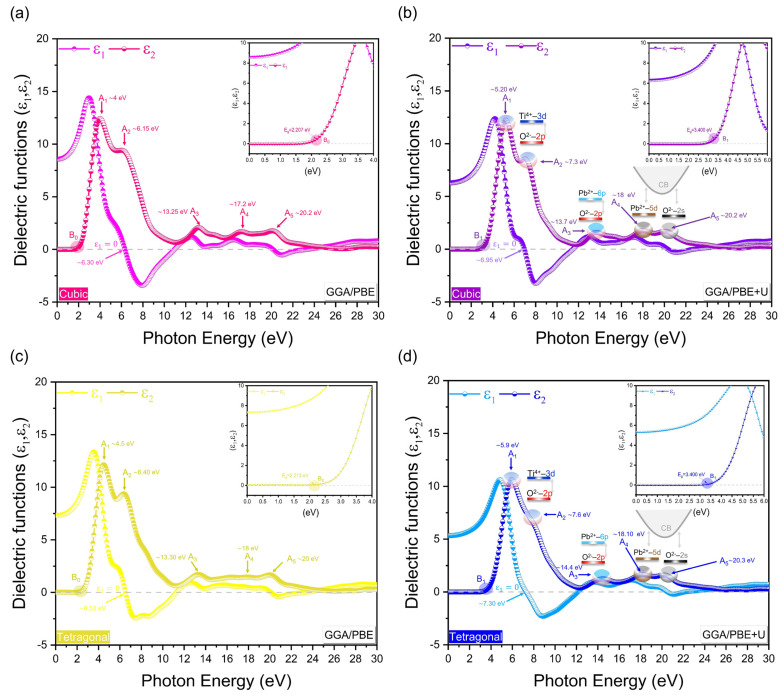
Imaginary/real parts of the dielectric function of PbTiO_3_ without and with Hubbard correction (**a**,**b**) for the cubic phase and (**c**,**d**) for the tetragonal phase.

The optical properties of materials have demonstrated themselves to be a valuable means of understanding and confirming the results obtained from the investigation of electronic properties. The following section systematically compares the optical properties of the cubic and tetragonal phases of PbTiO_3_, with and without the Hubbard U correction, with the existing experimental findings. Figure 9 shows the imaginary and real parts of the dielectric function of PbTiO_3_ with and without the Hubbard U correction. We will start by discussing the imaginary portion of the dielectric function, which is tightly related to the electronic band structure and shows the light absorption in the material. Using only the GGA/PBE approximation, the *ε*_2_ curve for the cubic and tetragonal phases indicates an energy of ~2.207 eV and ~2.213 eV, respectively, at the *B*_0_ point (see inset of Figure 9a,c), which is representative of the gap energy of PbTiO_3_. The Hubbard U potential correction to the cubic and tetragonal phases results in a shift of the CB toward higher energies of 1.193 eV and 1.187 eV, respectively. This reproduces the experimental band gap of 3.40 eV at the *B*_1_ point [14,15] (see inset of Figure 9b,d) and corrects the systematic discrepancy of the GGA approximation. From Figure 9a–d, the critical peaks of the optical *ε*_2_ spectra are denoted by *A*_1_, *A*_2_, *A*_3_, *A*_4_ and *A*_5_, all of which are shifted after the application of the U-correction to higher energies. The transition from O–2p (VBM) to Ti–3d (CBM) orbitals is mostly represented by the *A*_1_ and *A*_2_ peaks, whereas the transition from O–2p (VBM) to Pb–6p (CB) orbitals is primarily represented by the *A*_3_ peak. Moreover, the optical peaks *A*_4_ and *A*_5_ are associated with internal electronic excitation transitions of the Pb–5d and O–2s orbitals near VB to the CB semi-core states. It is necessary to note that each peak in *ε*_2_ is not always connected to a single interband transition because the band structure might contain many direct or indirect transitions with the same energy corresponding to the same peak. Our obtained *ε*_2_ data computed by GGA/PBE + U in the broad energy ranges agree with the currently available experimental findings of the cubic and tetragonal phases of PbTiO_3_ in the 0–6 eV range [41,42]; the other transitions were determined on the basis of our obtained results of densities of states (Figure 7) and agree similarly with the experimental and theoretical data of BaTiO_3_ and SrTiO_3_, which have been determined in broad energy ranges [43,44,45,46]. With respect to the real part of the dielectric function, similar results were observed to the imaginary part, where the calculated *ε*_2_(0) for the cubic (tetragonal) phase are about 6.30 eV (6.53 eV) and 6.95 eV (7.30 eV), respectively, in the case of GGA/PBE and GGA/PBE + U.

**Figure 10:**
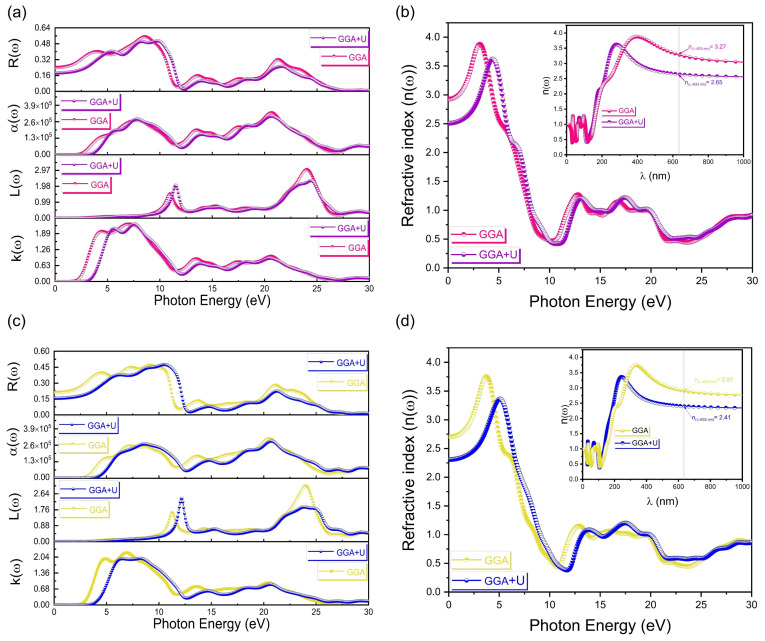
(**a**,**c**) Extinction coefficient, energy loss function, optical absorption coefficient and reflectivity of the cubic and tetragonal phases of PbTiO_3_, respectively, without and with Hubbard correction, at different values of the incident photon energy. (**b**,**d**) The refractive index of the cubic and tetragonal phases of PbTiO_3_, respectively, without and with Hubbard correction, at different values of both the incident photon energy and wavelengths.

With and without the Hubbard U correction, the calculated results for the energy dependence of the extinction coefficient *k*(*ω*), energy loss function *L*(*ω*), optical absorption coefficient *α*(*ω*) and reflectivity *R*(*ω*) of the cubic and tetragonal phases of PbTiO_3_ are shown in Figure 10a,c. As we can see, after the addition of the Hubbard U correction to the GGA approximation, a shift in the optical property spectrum toward higher energy is observed, which leads to correcting and increasing the gap energy of PbTiO_3_. From Figure 10b,d, the refractive index *n*(*ω*) shows the same behavior after applying the Hubbard U correction.

With respect to the wavelengths (see inset of Figure 10b,d), in order to establish a comparison between our computations and the experimental data, the refractive index of the cubic (tetragonal) phase is 3.27 (2.91) using only the GGA/PBE approximation, and becomes 2.65 (2.41) after applying the Hubbard U correction at the 633 nm wavelength. These results obtained with the Hubbard U correction agree with available experimental findings of refractive indices for sol–gel PbTiO_3_ films [15] and for metalorganic chemical vapor deposition (MOCVD) PbTiO_3_ films [6,47,48] by different techniques (Table 5). Based on an earlier examination of the optical characteristics, the Hubbard U potential adjustment was used, which, in turn, effectively offset the systematic error of the GGA approximation and produced results that are in accordance with the experimental data. On the other hand, the electronic analysis carried out for the cubic and tetragonal phases of PbTiO_3_ is supported by our optical characteristics.

## 4. Conclusions

In conclusion, this work presents a comprehensive overview of the crystal structure, chemical bonds, electronic and optical properties of the cubic and tetragonal phases of PbTiO_3_ using the GGA/PBE approximation with and without Hubbard U correction. First of all, we performed detailed calculations regarding geometry optimization to obtain the smallest difference across the experimental and optimized lattice parameters/volume, with the selection of the most appropriate methods of plane wave pseudopotential, k-points and cut-off energy, which validates the accuracy of our model. Secondly, the electronic investigations have shown that adding the Hubbard correction to the GGA/PBE approximation, with the well-selected values of Hubbard parameters for p- and d-orbitals, achieves an excellent result for the band gap (3.400 eV) of the cubic and tetragonal phases of PbTiO_3_ as compared to the experimental data. Additionally, the optimized chemical bonding analysis shows consistency with the experimental results, suggesting that the Pb–O and Ti–O bonds have a partial and strong covalent bonding nature, respectively. In addition, the application of the Hubbard U potential correction to the calculated optical properties could overcome the systematic inaccuracy of the GGA approximation. At the same time, studies of the complex dielectric function and other optical properties confirm the electronic calculations and offer excellent concordance with the actual results over a wide range of energies. Consequently, these results can be leveraged by scientists to expand the targeted scope of the cubic and tetragonal phases of PbTiO_3_.

## Figures and Tables

**Table 1:** Computation of the cell parameters, c/a tetragonality, volume and deviations (D) using the GGA/PBE approximation of both the cubic and tetragonal phases of PbTiO_3_ with appropriate k-point and cut-off energy values.

PbTiO_3_: Cubic Phase (*P*m$\bar{3}$m) (Pseudo-Potential Method: Norm-Conserving)									
**k-points**	**Cut-off (eV)**	**a_exp_ = b_exp_ = c_exp_ (Å)**	**a_opt_ = b_opt_ = c_opt_** **(Å)**		**D** **(%)**	**V_exp_** **(Å^3^)**		**V_opt_** **(Å^3^)**	**D** **(%)**
2 × 2 × 2	560	3.9702 ^[a]^	3.9700		0.0050	62.58 ^[a]^		62.573	0.0111
PbTiO_3_: Tetragonal phase (*P*4mm) (Pseudo-potential method: Norm-conserving)									
**k-points**	**Cut-off (eV)**	**Exp. lattice parameters (Å)**			**Opt. lattice parameters (Å)**			**D (%)**	
		**a_exp_ = b_exp_**	**c_exp_**	**c_exp_/a_exp_**	**a_opt_ = b_opt_**	**c_opt_**	**c_opt_/a_opt_**	**a, b**	**c**
2 × 2 × 2	480	3.904 ^[b]^	4.152 ^[b]^	1.063 ^[b]^	3.9295	4.1775	1.063	0.6489	0.6104

^[a]^ Experimental data from Ref. [12]; ^[b]^ Experimental data from Ref. [13]; a_exp_ and a_opt_ are the experimental and optimized lattice parameters, respectively. V_exp_ and V_opt_ are the experimental and optimized volume, respectively.

**Table 2:** Calculated lattice constants (a, b and c in Å) and c/a tetragonality using the GGA/PBE approximation, in comparison with available theoretical and experimental data of the cubic and tetragonal phases of PbTiO_3_.

Phases; Local Symmetry	This Calculation	Experimental Data	Previous Theoretical Results (DFT Functional)
			3.93 (LDA), 3.96 (PWGGA), 3.96 (PBE), 4.02 (BLYP), 3.93 (P3PW), 3.96 (P3LYP), 3.924 (HF) [25], 3.980 (GGA/PBE), 4.010 (GGA/RPBE), 3.977 (GGA/PW91), 3.899 (LDA/CA-PZ) [26], 3.970 (LDA) [38], and 3.987 (GGA/PBE) [39].
Cubic	(GGA/PBE)	3.9702 [12]	
(*P*m$\bar{3}$m); $O_{h}^{1}$	a = b = c = 3.9700	3.97 [13]	
		3.970 [36]	
Tetragonal (*P*4mm); $C_{4v}^{1}$	(GGA/PBE)	a = 3.9043, c = 4.1407, c/a = 1.060 [12].	a = 3.87, c = 4.07, c/a = 1.05 (LDA) [28], a = 3.872, and c/a = 1.041 (LDA); a = 3.834 and c/a = 1.221 (GGA/PBE) [40].
	a = b = 3.9295	a = 3.904, c = 4.152, c/a = 1.063 [13].	
	c = 4.1775	a = 3.905, c = 4.156, c/a = 1.064 [36].	
	c/a = 1.063	a = 3.896, c = 4.144, c/a = 1.063 [37].	

**Table 3:** Influence of the Hubbard U parameter values chosen for the O–2p, Ti–3d and Pb–5d orbitals on the gap energies of PbTiO_3_ using the GGA/PBE approximation while observing the minimum deviations (D). The cut-off energy/k-point values are 560 eV/2 × 2 × 2 and 480 eV/2 × 2 × 2 for the cubic and tetragonal phases, respectively.

Method	U_p_(O)	U_d_(Ti)	U_d_(Pb)	E_g_ (eV)		D (%)
				This Cal.	Exp Data.	
GGA/PBE	PbTiO_3_: Cubic phase (*P*m$\bar{3}$m) (Pseudo-Potential Method: Norm-Conserving)					
	0	0	0	2.207	-	-
	3.5	6	0	3.400	No exp. data for cubic phase	-
	3.5	6.5	0	3.395		-
	3.5	7	0	3.382		-
	4	5.5	0	3.431		-
	4.5	5	0	3.388		-
	5.5	4.5	0	3.395		-
	6.5	4	0	3.395		-
	7.5	3.5	0	3.387		-
	8	3.5	0	3.421		-
	8.5	3	0	3.396		-
	9	3	0	3.426		-
	10	2.5	0	3.390		-
	PbTiO_3_: Tetragonal phase (*P*4mm) (Pseudo-potential method: Norm-conserving)					
	0	0	0	2.213	-	-
	5	5.5	0	3.383	3.40 [14,15]	−0.502
	5	6	0	3.414		0.410
	5	7	0	3.400		0
	5	8	0	3.386		−0.413
	5.5	9	0	3.421		0.613
	5.5	9.5	0	3.390		−0.294
	6.5	10	0	3.416		0.468
	9.5	3.5	0	3.377		−0.681
	10	3.5	0	3.402		0.058

**Table 4:** Calculated Mulliken charges and effective valence charges of the cubic and tetragonal phases of PbTiO_3_ using the GGA/PBE + U approximation.

Species	Mulliken Charges (e)	Effective Valence Charges (e)
PbTiO_3_: Cubic phase (*P*m$\bar{3}$m)		
Pb	1.81	10.0
Ti	0.74	1.81
O	−0.85	0.00
PbTiO_3_: Tetragonal phase (*P*4mm)		
Pb	1.79	10
Ti	0.93	1.57
O_1_	−0.90	0.00
O_2_	−0.91	0.00
O_3_	−0.91	0.00

**Table 5:** Calculated refractive index values of the cubic and tetragonal phases of PbTiO_3_ without and with Hubbard correction in comparison with available experimental data.

Synthesis Method	Technique-DFT/Functional	Refractive Index	Ref.
Sol–gel PbTiO_3_	Transmittance	2.58	[15]
MOCVD PbTiO_3_ on SiTiO_3_	Ellipsometry	2.66	[6]
MOCVD PbTiO_3_ on SiTiO_3_	Prism coupling	2.675	[47]
MOCVD PbTiO_3_ on SiTiO_3_	Prism coupling	2.67	[48]
Primitive cell of PbTiO_3_ (cubic)	GGA/PBE	3.27	This Calc.
Primitive cell of PbTiO_3_ (cubic)	GGA/PBE + U	2.65	This Calc.
Primitive cell of PbTiO_3_ (tetragonal)	GGA/PBE	2.91	This Calc.
Primitive cell of PbTiO_3_ (tetragonal)	GGA/PBE + U	2.41	This Calc.

The experimental refractive indices were at the wavelength of 633 nm eV at RT.

## Data Availability

The data presented in this study are available from the corresponding author upon reasonable request.

## Supplementary Materials

The following supporting information can be downloaded at: https://www.mdpi.com/article/10.3390/ma16124302/s1, Table S1: Computation of the cell parameters, c/a tetragonality, volume and deviations using the GGA/PBE approximation of the cubic (*P*m$\bar{3}$m) and tetragonal (*P*4mm) phases of PbTiO_3_ as a function of the variation in the pseudopotential methods; the k-point and cut-off energy values were fixed at 2 × 2 × 2 and 500 eV, respectively; Table S2: Computation of the cell parameters and volume deviation using the GGA/PBE approximation of the cubic phase (*P*m$\bar{3}$m) of PbTiO_3_ as a function of the variation in the k-point values; the value of cut-off energy was set at 500 eV; Table S3: Computation of the cell parameters (a, c) and tetragonality c/a deviation using the GGA/PBE approximation of the tetragonal phase (*P*4mm) of PbTiO_3_ as a function of the variation in the k-point values; the value of cut-off energy was set at 500 eV; Table S4: Computation of the cell parameters and volume deviation using the GGA/PBE approximation of the cubic phase (*P*m$\bar{3}$m) of PbTiO_3_ as a function of the variation in the cut-off energy values; the k-point values were set at 2 × 2 × 2; Table S5: Computation of the cell parameters (a, c) and tetragonality c/a deviation using the GGA/PBE approximation of the tetragonal phase (*P*4mm) of PbTiO_3_ as a function of the variation in the cut-off energy values; the k-point values were set at 2 × 2 × 2; Table S6: Hubbard U parameter values chosen for the O–2p, Ti–3d and Pb–5d orbitals of the cubic phase (*P*m$\bar{3}$m) of PbTiO_3_ using the GGA/PBE approximation; Table S7: Hubbard U parameter values chosen for the O–2p, Ti–3d and Pb–5d orbitals of the tetragonal phase (*P*4mm) of PbTiO_3_ using the GGA/PBE approximation.
