# Supplementary material for: Overview of the Structural, Electronic and Optical Properties of the Cubic and Tetragonal Phases of PbTiO3 by Applying Hubbard Potential Correction
Source: Materials (Basel). 2023 Jun 10;16(12):4302. doi: 10.3390/ma16124302 (PMC10304663; doi:10.3390/ma16124302)
Supplement: Supplementary file 1 [file materials-16-04302-s001.zip › materials-2412242-supplementary.pdf]

materials

MDPI

Supplementary Materials for

Overview of the structural, electronic and optical properties of the cubic and tetragonal phases of $PbTiO_3$ by applying Hubbard potential correction

Issam Derkaoui [1], Mohamed Achehboune [2], Roberts I. Eglitis [3], Anatoli I. Popov [3,*] and Abdellah Rezzouk [1]

[1] Laboratory of Solid State Physics, Faculty of Sciences Dhar El Mahraz, University Sidi Mohammed Ben Abdellah, P.O. Box 1796, Atlas Fez 30000, Morocco; derkaouissam@gmail.com (I.D.); rezzouk@yahoo.fr (A.R.)
[2] Laboratoire de Physique du Solide, Namur Institute of Structured Matter, University of Namur, Rue de Bruxelles 61, 5000 Namur, Belgium; achehboune.mohamed01@gmail.com
[3] Institute of Solid State Physics, University of Latvia, 8 Kengaraga Str., LV1063 Riga, Latvia; rieglitis@gmail.com
* Correspondence: popov@latnet.lv

Contents

1. Geometry optimization

1.1 Appropriate pseudopotential methods

1.2 Appropriate ***k-points*** *and cut-off energy*

2. Hubbard U correction

References

1. Geometry Optimization

Further simulations were realized using different plane wave pseudopotential methods, using GGA with the PBE function as implemented in the CASTEP code.

1.1 Appropriate pseudopotential methods

Table S1 shows the lattice parameters, c/a tetragonality and volume calculated by the GGA/PBE approximation with different pseudopotential methods for the cubic ($Pm\bar{3}m$) and tetragonal ($P4mm$) phases of $PbTiO_3$. For the cubic phase, when we compare with the experimental data [1], the relative deviations of lattice parameters (volume) between the calculated structure parameters and the experimental values are about 0. 060% (0.188%) for the ultrasoft, 0.075% (0.227%) for the OTGF ultrasoft, 0.256% (0.770%) for the OTGF norm-conserving and 0.055% (0.166%) for the norm-conserving pseudopotential methods. On the other hand, for the tetragonal phase ($P4mm$) of $PbTiO_3$, the results presented in Table 1 show that the optimized lattice parameters a, c, and the tetragonality c/a provide minimal deviation from the experiment [2] and this is achieved by the norm-conserving pseudopotential method. Hence, the lattice parameters for both the cubic and tetragonal phases of $PbTiO_3$ calculated by norm-conserving pseudopotential method display the closest relative deviation to the experimental value.

Table S1. Computation of the cell parameters, c/a tetragonality, volume and deviations using the GGA/PBE approximation of the cubic ($Pm\bar{3}m$) and tetragonal ($P4mm$) phases of $PbTiO_3$ as a function of the variation of the pseudopotential methods; The **k-points** and cut-off energy values were fixed at 2×2×2 and 500 eV, respectively.

$PbTiO_3$: Cubic phase ($Pm\bar{3}m$) [**k-points**: 2×2×2; **Cut-off**: 500 eV]						
**Pseudo-potential methods**	**$a_{exp}=b_{exp}=c_{exp}$ (Å)**	**$a_{opt}=b_{opt}=c_{opt}$ (Å)**	**Deviation (%)**	**V_{exp} (Å^3)**	**V_{opt} (Å^3)**	**Deviation (%)**
Ultrasoft	3.9702[a]	3.9726	0.0604	62.58[a]	62.698	0.1882
OTFG ultrasoft		3.9732	0.0755		62.722	0.2267
OTFG norm-conserving		3.9804	0.2562		63.066	0.7706
**Norm-conserving**		**3.9724**	**0.0553**		**62.684**	**0.1659**

$PbTiO_3$: Tetragonal phase ($P4mm$) [**k-points**: 2×2×2; **Cut-off**: 500 eV]								
**Pseudo-potential methods**	**Exp. lattice parameters (Å)**			**Opt. lattice parameters (Å)**			**Deviation (%)**	
	**$a_{exp}=b_{exp}$**	**c_{exp}**	**c_{exp}/a_{exp}**	**$a_{opt}=b_{opt}$**	**c_{opt}**	**c_{opt}/a_{opt}**	**a, b**	**c**
Ultrasoft	3.904[b]	4.152[b]	1.063[b]	3.9853	3.9775	0.998	2.0399	-4.3871
OTFG ultrasoft				3.9927	3.9847	0.997	2.2215	-4.1985
OTFG norm-conserving				3.9802	4.2893	1.077	1.9144	3.2009
**Norm-conserving**				**3.9619**	**4.1613**	**1.050**	**1.4614**	**0.2234**

[a] Experimental data from Ref. [1]

[b] Experimental data from Ref. [2]

a_{exp} and a_{opt} are the experimental and optimized lattice parameters, respectively.
V_{exp} and V_{opt} experimental and optimized volume, respectively.

Table S2. Computation of the cell parameters and volume deviation using the GGA/PBE approximation of cubic phase ($Pm\bar{3}m$) of $PbTiO_3$ as a function of the variation of the **k-points** values; The value of cut-off energy was set at 500 eV.

$PbTiO_3$: ($Pm\bar{3}m$; O_h^1) [**Pseudo-potential method**: Norm-conserving]							
**Method**	**k-points**	**$a_{exp}=b_{exp}=c_{exp}$ (Å)**	**$a_{opt}=b_{opt}=c_{opt}$ (Å)**	**Deviation (%)**	**V_{exp} (Å^3)**	**V_{opt} (Å^3)**	**Deviation (%)**
GGA/PBE	1×1×1	3.9702[a]	failed	-	62.58[a]	failed	-
	**2×2×2**	3.9702[a]	**3.9724**	**0.0553**	62.58[a]	**62.684**	**0.1659**
	3×3×3	~	3.9478	-0.5674	~	61.529	-1.7081
	4×4×4	~	3.9728	0.0654	~	62.707	0.2025
	5×5×5	~	3.9632	-0.1766	~	62.254	-0.5236
	6×6×6	~	3.9570	-0.3335	~	61.962	-0.9973
	7×7×7	~	3.9672	-0.0756	~	62.438	-0.2274
	8×8×8	~	3.9630	-0.1816	~	62.242	-0.5430
	9×9×9	3.9702[a]	3.9603	-0.2499	62.58[a]	62.115	-0.7486

Table S3. Computation of the cell parameters (a, c) and tetragonality c/a deviation using the GGA/PBE approximation of tetragonal phase (*P*4mm) of $PbTiO_3$ as a function of the variation of the **k-points** values; The value of cut-off energy was set at 500 eV.

PbTiO3: Tetragonal phase (*P*4mm) [**Pseudo-potential method**: Norm-conserving]									
**Method**	**k-points**	**Exp. lattice parameters (Å)**			**Opt. lattice parameters (Å)**			**Deviation (%)**	
		**a_{exp}=b_{exp}**	**c_{exp}**	**c_{exp}/a_{exp}**	**a_{opt}=b_{opt}**	**c_{opt}**	**c_{opt}/a_{opt}**	**a, b**	**c**
GGA/PBE	1×1×1	3.904[b]	4.152[b]	1.063[b]	failed	failed		-	-
	2×2× 1	~	~	~	3.7732	5.3092	1.407	-3.4665	21.7961
	**2×2×2**	3.904[b]	4.152[b]	1.063[b]	**3.9619**	**4.1613**	**1.050**	**1.4614**	**0.2234**
	2×2× 3	~	~	~	3.9671	4.2049	1.059	1.5905	1.2580
	3×3×3	~	~	~	3.8258	4.8839	1.276	-2.0440	14.9859
	4×4×4	~	~	~	3.8433	4.9440	1.286	-1.5793	16.0194
	5×5×5	~	~	~	3.8474	4.9364	1.283	-1.4711	15.8901
	6×6×6	~	~	~	3.8396	4.9437	1.287	-1.6772	16.0143
	7×7×7	~	~	~	3.8464	4.9363	1.283	-1.4975	15.8884
	8×8×8	~	~	~	3.8436	4.9379	1.284	-1.5714	15.9156
	9×9×9	3.904[b]	4.152[b]	1.063[b]	3.8413	4.9495	1.288	-1.6322	16.1127

*1.2. Appropriate **k-points** and cut-off energy*

In an attempt to obtain the best minimum deviation from the experimental and optimized lattice parameters and volume of the cubic (*P*m$\overline{3}$m) and tetragonal (*P*4mm) phases of $PbTiO_3$, we will investigate the most convenient **k-points** and cut-off energy values. The primitive cell parameters a = b = c = 3.9702 Å (α = 90° ; volume = 62.58 Å) [1] and a = b = 3.904 Å, c = 4.152 Å (α = 90° ; volume = 63.28 Å; tetragonality c/a = 1.063) [2] were originally used for constructing the cubic (*P*m$\overline{3}$m) and tetragonal (*P*4mm) phases of $PbTiO_3$, respectively. Initially, by keeping the energy cut-off value constant (e.g. 500 eV), we will change the grid values (**k-points**) (see Tables S2-S3). Finally, after getting an appropriate value of **k-points**, we will maintain this value constant and vary the cut-off energy to get the most appropriate cut-off energy value (see Tables S4-S5).

For the cubic phase of $PbTiO_3$, the minimum deviation between the experimental and optimized both volume and lattice parameters occurs by setting the **k-points** to 2×2×2 (Table S2). Furthermore, following the use of several cut-off energies, we have found that the minimum deviation of the lattice parameters and volume is -0.005% and -0.011%, respectively, occurring for the cut-off energy value of 560 eV (Table S4). On the other hand, for the tetragonal phase of $PbTiO_3$, the minimum deviation between the experimental and optimized a and c lattice parameters, as well as the tetragonality c/a occurs by setting the **k-points** to 2×2×2 (Table S3). Furthermore, after using several cut-off energies, we found that the minimum deviation of a and c for the cut-off energy value of 480 eV (Table S5), which is less than 0.65%. These outcomes highlighted that the deviation in lattice parameters between computed and standard data [1, 2] occurred to be less than 0.0051% and 0.65% for the cubic (*P*m$\overline{3}$m) and tetragonal (*P*4mm) phases of $PbTiO_3$, respectively, which allows us to investigate the performance and diversity of structural parameters computed by the GGA/PBE approximation. Hence, the cut-off and **k-points** values selected after the optimization are in good agreement and proves the validity of our model, together with the smallest relative deviation from the experimental data.

Table S4. Computation of the cell parameters and volume deviation using the GGA/PBE approximation of cubic phase ($Pm\overline{3}m$) of $PbTiO_3$ as a function of the variation of the cut-off energy values; The **k-points** values were set at 2×2×2.

$PbTiO_3$: **(**$Pm\overline{3}m$; O_h^1) [**Pseudo-potential method**: Norm-conserving]							
**Method**	**Cut-off (eV)**	$a_{exp}=b_{exp}=c_{exp}$ **(Å)**	$a_{opt}=b_{opt}=c_{opt}$ **(Å)**	**Deviation (%)**	V_{exp} **(Å³)**	V_{opt} **(Å³)**	**Deviation (%)**
GGA/PBE	200	3.9702[a]	4.5707	13.1380	62.58[a]	95.493	34.4664
	300	~	4.0067	0.9109	~	64.326	2.7142
	400	~	4.1999	5.4691	~	74.083	15.5271
	420	~	4.0138	1.0862	~	64.665	3.2243
	440	~	4.1014	3.1989	~	68.991	9.2925
	460	~	3.9844	0.3563	~	63.256	1.0686
	480	~	3.9270	-1.1000	~	60.560	-3.3355
	490	~	3.9739	0.0931	~	62.757	0.2820
	500	~	3.9724	0.0553	~	62.684	0.1659
	510	~	3.9731	0.0729	~	62.720	0.2232
	520	~	3.9731	0.0729	~	62.721	0.2248
	540	~	3.9733	0.0780	~	62.729	0.2375
	550	~	3.9580	-0.3082	~	62.008	-0.9224
	**560**	3.9702[a]	**3.9700**	**-0.0050**	62.58[a]	**62.573**	**-0.0111**
	570	~	3.9693	-0.0226	~	62.540	-0.0639
	580	~	3.9683	-0.0478	~	62.490	-0.1440
	600	~	3.9584	-0.2981	~	62.026	-0.8931
	700	~	3.9444	-0.6540	~	61.368	-1.9749
	800	~	3.9406	-0.7511	~	61.193	-2.2666
	900	3.9702[a]	3.9399	-0.7690	62.58[a]	61.162	-2.3184

2. Hubbard Correction

To give a good prediction of the electronic structure, we performed a sequence of probabilities associated with the choice of Hubbard potential (O-2p, Ti-3d and Pb-5d) for both the cubic and tetragonal phases of $PbTiO_3$, as shown in Tables S6-S7. As we can see, the gap energy is not significantly influenced even by varying the Hubbard potential values for Pb-5d electrons, due to its position in the internal electronics close to the valence band. Therefore, we adopted the modified Hubbard potential U for Ti-3d and O-2p electrons in order to accurately describe the electronic structures. For the tetragonal phase, the evaluation presented in Table S7 highlights the various probabilities of the chosen values of the Hubbard U parameters (Ti-3d and O-2p), which gave us band gaps in fairly good agreement with those obtained experimentally (3.40 eV) [3, 4]. Since the band gap of the cubic phase of $PbTiO_3$ is experimentally unknown, we performed a sequence of probabilities associated with the choice of the Hubbard potential (O-2p, Ti-3d) in comparison with the tetragonal phase (Table S6).

Table S5. Computation of the cell parameters (a, c) and tetragonality c/a deviation using the GGA/PBE approximation of tetragonal phase (P4mm) of $PbTiO_3$ as a function of the variation of the cut-off energy values; The **k-points** values were set at 2×2×2.

PbTiO$_3$: Tetragonal phase (*P*4mm) [**Pseudo-potential method**: Norm-conserving]									
**Method**	**Cut-off (eV)**	**Exp. lattice parameters (Å)**			**Opt. lattice parameters (Å)**			**Deviation (%)**	
		$a_{exp}=b_{exp}$	c_{exp}	c_{exp}/a_{exp}	$a_{opt}=b_{opt}$	c_{opt}	c_{opt}/a_{opt}	**a, b**	**c**
GGA/PBE	200	3.904[b]	4.152[b]	1.063[b]	4.2868	5.0214	1.171	8.9297	17.3138
	300	~	~	~	3.9829	5.5295	1.388	1.9809	24.9118
	400	~	~	~	4.0883	4.4137	1.079	4.5079	5.9292
	420	~	~	~	4.0325	4.2251	1.047	3.1866	1.7301
	440	~	~	~	4.0292	4.1636	1.033	4.0292	0.2786
	460	~	~	~	4.0204	4.0211	1.000	2.8952	-3.2553
	470	~	~	~	3.9774	4.2218	1.061	1.8454	1.6533
	**480**	3.904[b]	4.152[b]	1.063[b]	**3.9295**	**4.1775**	**1.063**	**0.6489**	**0.6104**
	490	~	~	~	3.9720	4.2188	1.062	1.7119	1.5833
	500	~	~	~	3.9619	4.1613	1.050	1.4614	0.2234
	510	~	~	~	3.8698	5.1236	1.323	-0.8837	18.9632
	520	~	~	~	3.9651	4.1586	1.048	1.5409	0.1587
	530	~	~	~	3.8378	5.2892	1.378	-1.7249	21.500
	540	~	~	~	3.8208	5.2449	1.372	-2.1775	20.8373
	560	~	~	~	3.8501	5.1762	1.344	-1.3999	19.7867
	580	~	~	~	3.8492	5.1586	1.340	-1.423	19.5130
	600	~	~	~	3.9790	4.0864	1.026	1.8848	-1.6053
	700	~	~	~	3.9704	3.9608	0.997	1.6723	-4.8273
	800	~	~	~	3.9609	3.9557	0.998	1.4365	-4.9624
	900	3.904[b]	4.152[b]	1.063[b]	3.9633	3.9565	0.998	1.4962	-4.9412

Table S6. Hubbard U parameter values chosen for the O-2p, Ti-3d and Pb-5d orbitals of the cubic phase ($Pm\bar{3}m$) of $PbTiO_3$ using the GGA/PBE approximation.

**U(O-2p)**	**U(Ti-3d)**	**U(Pb-5d)**	**E_g [eV]**	
			**This calculation**	**Experimental data**
PbTiO$_3$: (*P*m$\bar{3}$m; O_h^1) [**Pseudo-potential method**: Norm-conserving]; **k-points**: 2×2×2; **cut-off**: 560 eV]				
**0**	**0**	**0**	**2.207**	-
0	2.5	0	2.392	-
0	3	0	2.443	-
0	3	2/3	2.442	-
0	3	4	2.443	-
0	3	6	2.443/	-
0	5	0	2.433	-

0	7	0	2.400	-
0	9	0	2.335	-
2.5	4	0	2.986	No experimental data for cubic phase
2.5	6	0	3.128	
2.5	8	0	3.077	
3	5	0	3.210	
3	6	0	3.272	
3	7	0	3.235	
3.5	5.5	0	3.372	
**3.5**	**6**	**0**	**3.400**	
**3.5**	**6.5**	**0**	**3.395**	
**3.5**	**7**	**0**	**3.382**	
4	5	0	3.334	
**4**	**5.5**	**0**	**3.431**	
4	6	0	3.509	
**4.5**	**5**	**0**	**3.388**	
4.5	5.5	0	3.488	
5	4.5	0	3.346	
**5**	**5**	**0**	**3.440**	
5	5.5	0	3.542	
5.5	4	0	3.306	
**5.5**	**4.5**	**0**	**3.395**	
5.5	5	0	3.492	
6	4	0	3.351	
**6**	**4.5**	**0**	**3.442**	
6	5	0	3.540	
6.5	3.5	0	3.308	
**6.5**	**4**	**0**	**3.395**	
6.5	4.5	0	3.488	
7	3.5	0	3.349	
**7**	**4**	**0**	**3.437**	
7	4.5	0	3.531	
**7.5**	**3.5**	**0**	**3.387**	
**7.5**	**4**	**0**	**3.477**	
7.5	4.5	0	3.571	
8	3	0	3.337	
**8**	**3.5**	**0**	**3.421**	
8	4	0	3.514	
8.5	2.5	0	3.313	
**8.5**	**3**	**0**	**3.396**	
**8.5**	**3.5**	**0**	**3.456**	

8.5	4	0	3.550
9	2.5	0	3.338
**9**	**3**	**0**	**3.426**
9	3.5	0	3.517
9.5	2	0	3.284
9.5	2.5	0	3.364
**9.5**	**3**	**0**	**3.450**
10	2	0	3.308
**10**	**2.5**	**0**	**3.390**
10	3	0	3.480

Table S7. Hubbard U parameter values chosen for the O-2p, Ti-3d and Pb-5d orbitals of the tetragonal phase (P4mm) of $PbTiO_3$ using the GGA/PBE approximation.

**U(O-2p)**	**U(Ti-3d)**	**U(Pb-5d)**	**E_g [eV]**	
			**This calculation**	**Experimental data**
$PbTiO_3$: (*P*4mm; C_{4v}^1) [**Pseudo-potential method**: Norm-conserving]; **k-points**: 2×2×2; **cut-off**: 480 eV]				
**0**	**0**	**0**	**2.213**	-
0	2.5	0	2.465	-
0	3	0	2.412	-
0	3	2	2.442	-
0	3	4	2.488	-
0	3	6	2.484	-
0	5	0	2.446	-
0	7	0	2.358	-
0	9	0	2.286	-
4	6	0	3.233	-
4	7	0	3.172	-
**5**	**5.5**	**0**	**3.383**	3.40 [3, 4]
**5**	**6**	**0**	**3.414**	3.40 [3, 4]
**5**	**7**	**0**	**3.400**	3.40 [3, 4]
**5**	**8**	**0**	**3.386**	3.40 [3, 4]
5.5	4.5	0	3.141	-
5.5	5	0	3.339	-
5.5	6	0	3.495	-
5.5	8	0	3.456	-
**5.5**	**9**	**0**	**3.421**	3.40 [3, 4]
**5.5**	**9.5**	**0**	**3.390**	3.40 [3, 4]
5.5	10	0	3.283	-
6	5	0	3.257	-
6	5.5	0	3.351	-

6	6	0	3.446	-
6	9.5	0	3.479	-
6	10	0	3.199	-
6.5	5.5	0	3.501	-
6.5	6	0	3.486	-
6.5	7	0	3.669	-
6.5	9	0	3.599	-
6.5	9.5	0	3.529	-
**6.5**	**10**	**0**	**3.416**	3.40 [3, 4]
7	5	0	3.330	-
7	5.5	0	3.539	-
7	6	0	3.642	-
7	10	0	3.461	-
7.5	5	0	3.360	-
7.5	5.5	0	3.574	-
7.5	6	0	3.685	-
7.5	10	0	3.512	-
8	4	0	3.224	-
8	4.5	0	3.481	-
8	5	0	3.568	-
8	10	0	3.575	-
8.5	4	0	3.253	-
8.5	4.5	0	3.499	-
8.5	5	0	3.611	-
8.5	10	0	3.641	-
9	4	0	3.278	-
9	5	0	3.448	-
9	10	0	3.693	-
**9.5**	**3.5**	**0**	**3.377**	3.40 [3, 4]
9.5	4	0	3.470	-
9.5	10	0	3.719	-
10	3	0	3.299	-
**10**	**3.5**	**0**	**3.402**	3.40 [3, 4]
10	4	0	3.494	-
10	10	0	3.760	-

References

1. Yoshiasa, A.; Nakatani, T.; Nakatsuka, A.; Okube, M.; Sugiyama, K.; Mashimo, T. High-Temperature Single-Crystal X-Ray Diffraction Study of Tetragonal and Cubic Perovskite-Type $PbTiO_3$ Phases. *Acta Crystallogr B Struct Sci Cryst Eng Mater* **2016**, *72*, 381–388, doi:10.1107/S2052520616005114.
2. Shirane, G.; Pepinsky, R.; Frazer, B.C. X-Ray and Neutron Diffraction Study of Ferroelectric $PbTiO_3$. *Acta Cryst* **1956**, *9*, 131–140, doi:10.1107/S0365110X56000309.

3. Robertson, J. Band Offsets of Wide-Band-Gap Oxides and Implications for Future Electronic Devices. *J. Vac. Sci. Technol. B* **2000**, *18*, 1785, doi:10.1116/1.591472.
4. Peng, C.H.; Chang, J.-F.; Desu, S.B. Optical Properties of PZT, PLZT, and PNZT Thin Films. *MRS Proc.* **1991**, *243*, 21, doi:10.1557/PROC-243-21.

Disclaimer/Publisher's Note: The statements, opinions and data contained in all publications are solely those of the individual author(s) and contributor(s) and not of MDPI and/or the editor(s). MDPI and/or the editor(s) disclaim responsibility for any injury to people or property resulting from any ideas, methods, instructions or products referred to in the content.
